# The Effect of Simulated Visual Field Loss on Optokinetic Nystagmus

**DOI:** 10.1167/tvst.9.3.25

**Published:** 2020-02-21

**Authors:** Soheil M. Doustkouhi, Philip R. K. Turnbull, Steven C. Dakin

**Affiliations:** 1 School of Optometry & Vision Science, University of Auckland, Auckland, New Zealand; 2 New Zealand National Eye Centre, University of Auckland, Auckland, New Zealand; 3 UCL Institute of Ophthalmology, University College London, London, UK

**Keywords:** visual field, eye movements, peripheral vision

## Abstract

**Purpose:**

Assessment of functional vision across the visual field is hampered by a reliance on patients' subjective judgement of the presence of a stimulus, and the accompanying demands (time and attention) this places on them. As a first step toward determining whether an objective measure of an involuntary eye movement (optokinetic nystagmus [OKN]) could provide an objective measure of field loss, we determined how various measures of OKN depend on the extent of simulated visual field loss (SVFL).

**Methods:**

We used infrared eye-tracking to measure the eye movements of 16 healthy participants viewing horizontally translating 2-dimensional noise patterns over trials of varying contrasts and different levels of SVFL. We quantified the strength of OKN by estimating the velocity of tracking eye movements compared to the stimulus (OKN gain). These measurements were made using an open-loop SVFL paradigm, where a varying amount of gaze-contingent peripheral stimuli was occluded.

**Results:**

Full-field stimulation led to an average OKN gain of 0.92 ± 0.15. This value fell steadily with increasing SVFL to a value of 0.38 ± 0.20 when the periphery was not stimulated at all (i.e., the stimulus was a 5-deg. diameter foveal patch). We note considerable individual variation in OKN gain in all conditions.

**Conclusions:**

Measuring the extent of visual field loss using an objective measure of OKN gain is feasible.

**Translational Relevance:**

Simulated visual field loss reduces optokinetic nystagmus, but further refinement of this technique would be required to overcome individual differences and to pick up clinically relevant field defects.

## Introduction

Glaucoma is a progressive eye disease that causes loss of retinal ganglion cells RGCs^1^ and is the leading cause of irreversible blindness worldwide.^2^ Vision loss typically starts from the mid-periphery of the retina and may spread across the entire visual field.^3^ The function of the visual field is typically measured using white-on-white *standard automated perimetry* (SAP), which relies on the patient's ability to report a slight luminance difference of a stimulus against a uniform background.^4^ Although SAP remains the gold standard for assessing the integrity of the visual field,^5^ its sensitivity and accuracy are limited by several factors. These include the experience of the examiner,^6^ the time of day when testing is conducted,^6^ refractive blur,^7^^,^^8^ severity of field loss,^9^ and the specific instructions given to patients.^10^ Alternatives to SAP include short-wavelength automated perimetry, which probes the integrity of the short-wavelength cone system,^4^ frequency doubling, an illusion based on perceived rate of flicker,^11^ and motion automated perimetry, which attempts to isolate dysfunctional magnocellular processing using moving targets.^12^ These approaches can all detect functional vision loss arising from glaucoma at least as early as SAP.^12^^–^^15^

A variety of objective methods have been presented as alternatives to SAP. These include pattern ERG,^16^ multifocal ERG,^17^^,^^18^ and multifocal VEP,^19^^,^^20^ all of which require a high level of operator expertise and patient compliance and cooperation.^21^ It has also been proposed that eye movements can provide an objective assessment of a patient's visual status. For example, in eye movement perimetry (EMP) a patient's saccades to peripherally presented targets are used to quantify visual function across the field.^22^^–^^27^ Using this paradigm, glaucoma patients require more contrast to initiate saccades, and those saccades tend to be less accurate than those made by controls.^27^

Saccades are voluntary eye movements so that EMP (like SAP) requires a level of cooperation from the patient. In contrast, optokinetic nystagmus (OKN) is a reflexive eye movement made in response to stimulus motion. It consists of periods of smooth tracking in the stimulus-direction, interspersed with saccades in the opposite direction. OKN serves to reduce “retinal slip” by partially stabilising the moving image on the retina. The strength of OKN is measured using *OKN gain*, the ratio of the velocity of the slow phase of OKN to the velocity of the stimulus. Another way of quantifying OKN is by classifying the pattern of eye movements (tracking and saccades) as being consistent or inconsistent with an optokinetic response to the stimulus direction. This measure can serve as a proxy for “seen” or “unseen” responses from patients, respectively. Note that because OKN is an involuntary reflex, this procedure reduces the level of cooperation required from patients.

Perimetric techniques measure *local visual dysfunction* by using stimuli that cover only a small portion of the visual field. OKN, on the other hand, requires relatively large stimuli.^28^ This means that detection of dysfunction arising from glaucoma would have to focus on quantifying a change in the optokinetic response to larger, global stimuli. Previous studies have used psychophysical motion coherence paradigms to show that glaucoma does compromise visual processing of global motion. Using full field (60- × 60-deg.) motion stimuli^29^^,^^30^ patients with glaucoma had motion thresholds 70% higher than the control group.^29^

In terms of quantifying how different parts of the visual field might contribute to OKN, several groups have used selective retinal stimulation to address this issue, typically using masks to selectively occlude different regions of the stimulus within either an open- or closed-loop eye-tracking paradigm.^28^^,^^31^^–^^36^ In open-loop experiments, the position of the mask is gaze-contingent, whereas in closed loop experiments the mask location is fixed. Such work has demonstrated that central vision plays a dominant role in the generation of OKN.^28^^,^^33^^–^^35^^,^^37^ However, measurement of OKN in patients with central scotomas indicates that peripheral retina also contributes to OKN.^32^^,^^38^^–^^40^ Here—as a first step toward developing objective measures of glaucomatous field loss based on eye-tracking—we sought to measure the effect of simulated visual field loss (SVFL) on OKN.

## Method

### Study Design

Experiment 1 examined the effect of SVFL on OKN gain. Experiment 2 investigated the effect of simulated contrast loss for stimuli presented at 2 levels of SVFL. The 2 experiments were completed in a single visit, in a randomized order for each participant, with a minimum 5-minute break between each. Total testing time was approximately 17 minutes per participant.

The experimental protocol and procedure were approved by the University of Auckland Human Research Ethics Committee (ref no. 019326). The experiment complied with the Declaration of Helsinki. Written consent was obtained from each participant, and participants were free to withdraw at any stage without giving a reason.

### Participants

Sixteen participants (21–50 years old, 9 females) took part in experiments to measure the OKN response of their right eyes. All participants either had had a recent eye examination (within 6 months) or, if they had not had a recent examination, were examined at the time of testing. Our exclusion criteria were amblyopia, or neurological disorders such as epilepsy. Fourteen of the 16 participants had normal or corrected-to-normal vision in the tested (right) eye. This meant that they either did not habitually require correction (5/14), wore their correction (3/14), or that their residual refractive error (at the 1-m test distance) was between −0.13 and +1.13D (6/14). Hyperopic participants in this group could accommodate to overcome low levels of residual refractive error. The remaining 2 participants (P2 & P3) did not wear their habitual optical correction because it interfered with eye-tracking, and these individuals had residual refractive errors of −0.50D (P3) and −0.75D (P2) tested at 1.0 m. Such a modest level of residual refractive error will have a negligible impact on the visibility of the patterns as we discuss in the Discussion section.

### Experimental Setup

Stimuli were presented on a 621 × 341 mm LCD monitor (S2817Q; Dell, Round Rock, TX, USA) with a 3840 × 2160 pixel resolution running at 60Hz. At the one-meter test distance, the display subtended 32- × 19-deg. and had a pixel density of 120 pixels per degree. The monitor was linearized in software based on measurements made with a photometer (LS100, Konica Minolta, Japan). Experiments were written on the stimulus computer in Matlab (version 2017b, MathWorks, Natick, MA) using Psychophysics Toolbox^41^ and the Eyelink Toolbox.^42^ Monocular eye movements were recorded on a separate computer, using an infrared eye-tracker (Eyelink 1000 Plus; SR Research, Kanata, Ontario, Canada) in remote mode, that is, without the use of a chin rest, at 500 Hz. Data were streamed to the stimulus computer over a direct ethernet connection. The eye-tracker stream was sampled at 60 Hz, using the sample immediately after the screen refresh. Light levels in the study room were low (5.8 lux), with windows covered and monitors (other than the stimulus monitor) rotated away from the participant to minimize distraction.

### Stimulus

**Figure 1.**
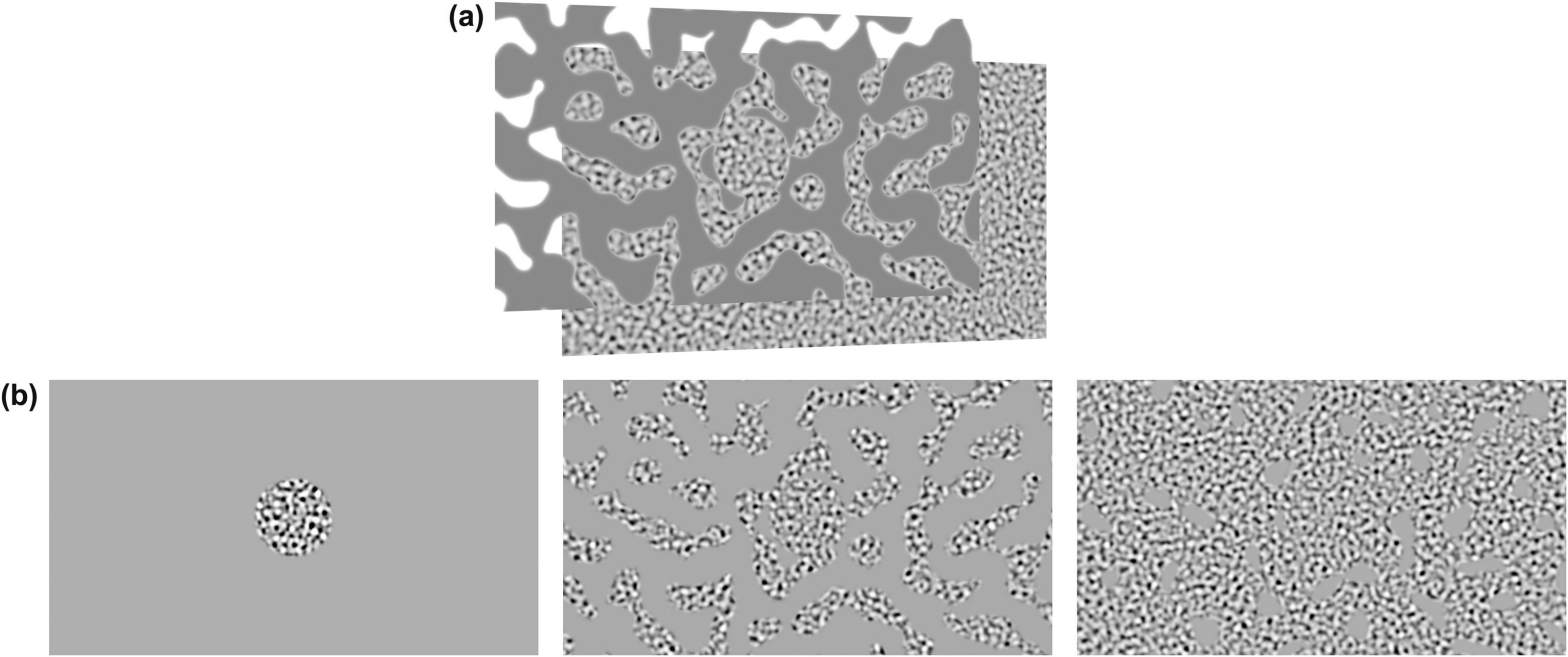
Stimulus generation. (a) The stimulus was constructed using two layers. The layer at the back is the isotropic noise carrier which drifted to the left or the right. The layer at the front is a masking image—note the carrier is visible through the transparent part—which moved with the patient's gaze. (b) The resulting stimulus after occluding the carrier with the mask. From left to right, either 3%, 39%, or 88% of the carrier is visible through the mask.

The stimulus was a spatial frequency filtered 2-dimensional random noise carrier, with a superimposed peripheral gray mask that occluded the carrier and served to simulate visual field loss (Fig. 1).

The carrier stimulus was generated in Matlab using 2-dimensional Gaussian noise that was filtered with a logGabor filter to contain a narrow range of spatial-frequencies^43^ and all orientations. Random fluctuations in local contrast across the stimulus were minimized using a demodulation technique described previously.^44^ The mean gray-level of the carrier was equal to the mid gray-level of the screen (60 cd/m^2^). To minimize any influence of the visual acuity of our participants, the stimulus was highly supra-threshold with a peak spatial-frequency of 1.3 c/deg, a bandwidth of 0.5 octaves and a contrast of 100% (in Experiment 1).

The gray mask (Fig. 1a) was also generated using logGabor filtered 2-dimensional Gaussian noise (0.26 c/deg, bandwidth 0.5 octaves), with a threshold imposed to binarize the stimulus. Values above the mean gray-level were set to the mean gray-level of the stimulus, and values below made transparent. A central disk (5-deg. diameter) within the mask of the gray mask was made transparent so that the carrier was always visible at fixation. The sharp edges of the mask were smoothed using a Gaussian blur kernel with a standard deviation of 4 pixels (2.4 arc min).

The mask shifted position in a gaze-contingent manner (i.e., it moved independently of the carrier). The carrier moved horizontally (left or right) at 10 deg/s, for a duration of 2 seconds. Stimulus direction was randomly stratified to ensure equal numbers of leftwards and rightwards trials. A gray screen appeared between trials to minimize any build-up of motion adaptation.

Experiment 1 had participants perform 8 trials at each of 9 different levels (3.3%, 15.3%, 27.6%, 39.5%, 51.6%, 63.8%, 75.8%, 88%, and 100%) of simulated visual field loss (i.e., 72 total trials). In this experiment, the carrier was presented at full contrast. Experiment 2 used just 2 levels of SVFL—either no loss (full field) or 50% loss—and modified the contrast of the carrier over seven log-spaced levels (1.6%, 3.1%, 6.3%, 12.5%, 25%, 50%, and 100%). Each stimulus was repeated 8 times (2 levels of SVFL × 7 contrasts × 8 trials = 112 trials).

### Procedure

We performed a 9-point eye-tracking calibration procedure for each participant before each experiment. During the test phase, participants were instructed to attempt to maintain fixation on the center of the screen, that is, “stare OKN.”^45^ For participants unfamiliar with the task, we ran a demo experiment before data collection that consisted of a sequence of 2-second trials that were run until the participant was comfortable with the task.

The main test consisted of a sequence of 2-second trials. After the presentation of the test stimulus the screen returned to mid-gray and participants were required to indicate the perceived direction (left or right) of the carrier using the computer keyboard. Participants then received immediate feedback for their response (to promote vigilance) through the color (green or red) of a rectangular frame around the edge of the screen. We used a peripheral frame to avoid providing an explicit fixation marker for subsequent trials. Trials were automatically repeated when less than 80% of possible eye-tracking samples were recorded (e.g., because the participant looked away).

### Quantifying OKN

To measure the strength of OKN, we quantified the velocity of participants’ tracking eye-movements relative to the velocity of the stimulus, using an automated method. We refer to this measure as *OKN gain*.

Eye movement data were analyzed offline after the experiment was complete. For ease of analysis, in trials with a leftward moving stimulus, eye position data were rotated 180°, so all eye movements were relative to a common stimulus direction. Eye-tracking data were first filtered to remove artefacts such as blinks. We did this by removing outlier where the estimated instantaneous pupillary area deviated by more than 2.75 × SD from the mean pupillary area for the trial. To be conservative and allow time for the eyetracker to accurately reacquire the eyes, we also removed 5 samples (83.3 ms = 5 × 16.7 ms of the 60 Hz sampled data) from either side of these samples. Such filtering necessarily breaks up the sequence of eye-tracking data, but we analyzed remaining data by simply joining any resulting subsequences within a trial's dataset.

**Figure 2.**
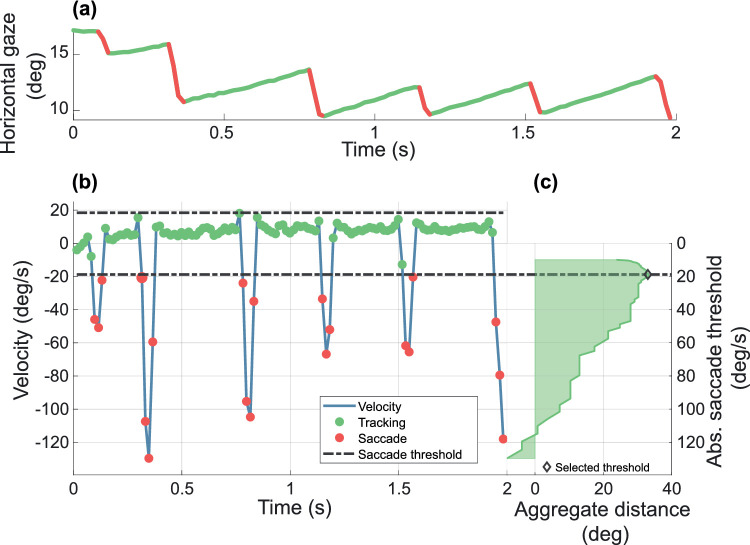
(a) Gaze position across a 2-second trial, classified as “tracking” (*green*) or “saccade” (*red*) using the procedure illustrated below. (b) Estimated eye velocity. *Dashed lines* indicate the velocity threshold. Velocities whose magnitude exceeded the magnitude of the threshold were classed as saccades (*red symbols*). (c) Adaptive selection of saccade threshold from a range of candidate thresholds. The plot shows the aggregate distance moved by the eye during tracking and saccade, for each candidate saccade-threshold. The threshold selected maximized this distance. Aggregate distance is the distance eye moved along with the stimulus minus the distance eye moved against the stimulus drifting direction.

To reduce noise in our estimates of eye position, we applied a second order Savitzky-Golay filter^46^ with a frame length of five samples. The derivative of eye position was used to calculate the instantaneous velocity of eye movements. We then classified all points as either “tracking” or “saccade”: using a range of candidate “saccadic thresholds” (the minimum velocity required for a datum to be classed as a saccade), we calculated the total distance the eye moved during tracking, minus that traveled during a saccade, and selected the threshold that maximized the aggregate distance (Fig. 2).^43^

To estimate OKN gain, we first accommodated the rise time for OKN by excluding data collected with the first 500 ms of each trial. We then fit estimated tracking velocities using a first-order generalized linear model with a slope of 0. The y-axis intercept of the fitted line is the estimated average tracking velocity of the eye during that trial. Finally, the gain of OKN is defined as the ratio between this estimated velocity and the velocity of the stimulus.

### Statistical Analysis

Unless otherwise stated, statistical analyses were conducted in Matlab. Our main outcome measure was OKN gain, versus the proportion of the stimulus that was visible (Experiment 1) or the contrast level (Experiment 2). We calculated the mean OKN gain for each participant over eight repetitions of each tested level. The mean OKN gain data across all participants for each condition (i.e., extent of SVFL in Experiment 1, contrast in Experiment 2) were fit with a Naka-Rushton function^47^ defined as:

$$\begin{array}{c} R=R_{max}\;\frac{L^{n}}{L^{n}}+{L_{50}}^{n}. \end{array}$$

Here *R* is the response (OKN gain), *R_max_* is the maximum response achieved, *L* is the stimulus level (SVFL or contrast), *L*_50_ is the stimulus level which elicits an OKN gain of 50% of *R_max_* and *n* is the exponent. We fit the mean OKN gain for each participant, and across all participants to estimate *R_max_*, *L*_50_ and *n*. To improve the fit of individual participants, we defined the upper boundary of *R_max_* as the observed maximum mean gain for each participant. Note that we settled on this as a good fit to our data after comparing other simpler fits, such as a log-linear fit to log (OKN-gain) versus SVFL.

In both experiments, we analyzed the mean OKN gain across condition to compute a repeated measure analysis of variance (ANOVA) (with a significance threshold of *P* < 0.05), using Mauchly's sphericity test with Greenhouse-Geisser correction, as required.

Monte-Carlo simulation (using data from Experiment 1) was used to determine the sensitivity and specificity of our method for detecting SVFL. We synthesized 800,000 samples (8 repetitions of 100,000 simulated participants repetitions) of predicted OKN gains across 64 synthesized VF extents (from 0.03 to full field). We then predicted OKN gains using the Naka-Rushton fit parameters from Experiment 1 and matched within-subject and between-subject variability (using standard deviations calculated from Experiment 1). Next, for determining the sensitivity and specificity of our method, we used a range of detection criteria and calculated the hit rate and false-positive rate for each level of visual field and criterion level. The range of cutoff criteria was from of *mean OKN gain*_min field_ – 3 × SD to *mean* OKN gain_max field_ + 3 × SD (mean OKN gain_min field_ and mean OKN gain_max field_ is the predicted OKN gain form Naka-Rushton fit for the 5-deg. central field and full field OKN respectively, and the *SD* is within-observer standard deviation). The false-positive and hit rates were used to plot ROC curves and corresponding d′ values.

## Results

### Experiment 1: Effect of Simulated Visual Field Loss (SVFL) on OKN Gain

**Figure 3.**
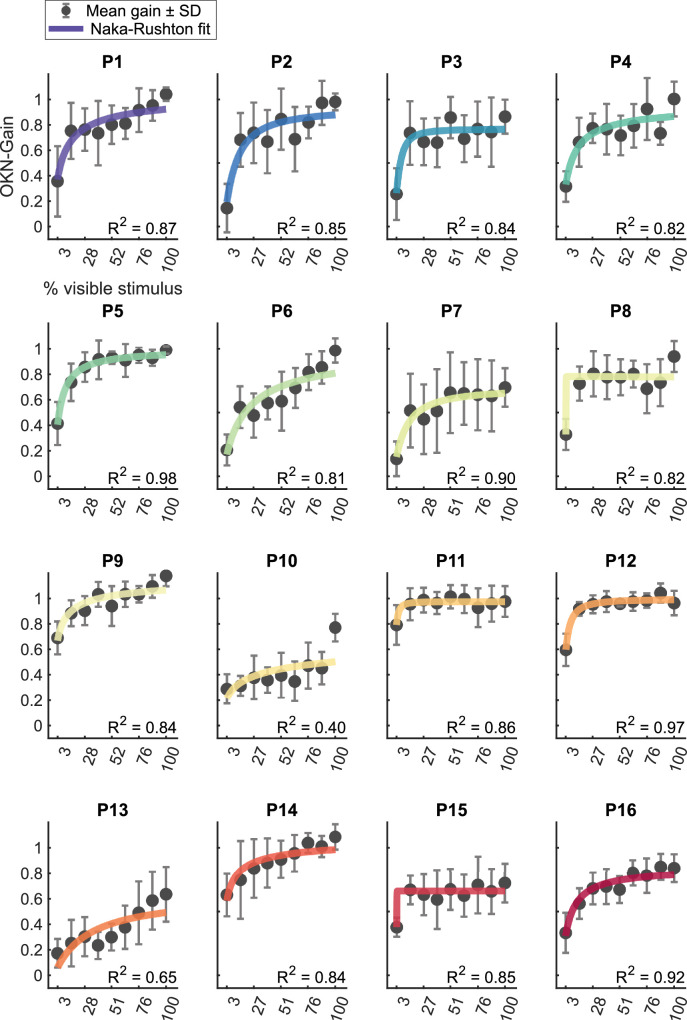
Estimated gain of the optokinetic response plot against the percentage of stimulus that was visible to our sixteen participants. Error bars indicate ±1 SD around the mean. Data have been fit with a Naka-Rushton function (described in “Statistical analysis”).

Figure 3 plots estimated OKN-gain against the percentage of visible-stimulus for our 16 participants. Visually, the data appear to be well captured by the Naka-Rushton fits, although we note considerable variation in the maximum OKN-response obtained and in the rate of increase of OKN with the percentage of visible stimulus. To determine whether the Naka-Rushton was a good fit to our data—compared to simpler fits with 2 free parameters—we compared adjusted *R*^2^ estimates of goodness-of-fit for the Naka-Rushton to a log-linear fit (of log-Gain, vs. linear-% visible stimulus) . The adjusted *R*^2^ for the Naka-Rushton fits were higher in 11 of 16 participants (with an average adjusted *R*^2^ across all participants of 0.722) compared to the log-linear fit (average adjusted *R*^2^ of 0.530). Naka-Rushton fits on individual data gave estimates of *R*_max_ of 0.80 ± 0.16 (mean ± 1 SD) and *L*_50_ of 0.087 ± 0.086.

**Figure 4.**
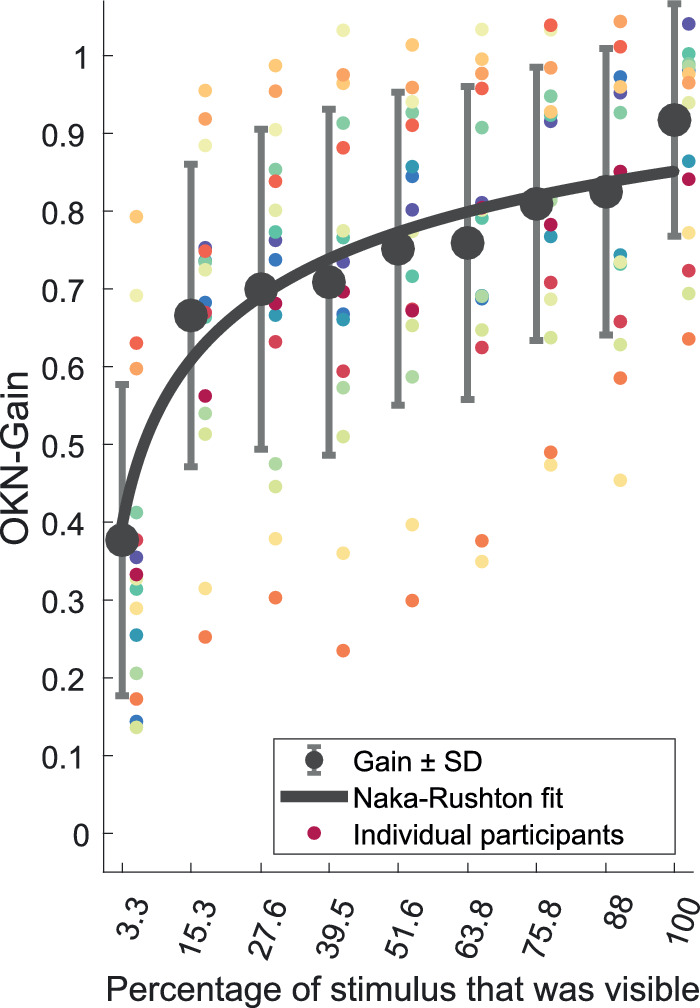
Results averaged across participants; OKN gain increases with the extent of the peripheral visual field that is stimulated. Small colored dots are the mean gain (across 8 trials) for each participant (Note that the color coding of each individual participant is consistent with Fig. 3). *Large* *gray* *circles* are the mean gain across participants. Mean values have been fit with a Naka-Ruston fit (*solid black line*). Error bars are ±1SD.

Pooling data across participants (Fig. 4), OKN gain was positively correlated with the spatial extent of the peripheral visual field, and ranged from 0.38 ± 0.20 measured with a 5-deg. diameter foveal stimulus-patch to 0.92 ± 0.15 measured with a full-field stimulus (*r* = 0.88, *P* = 0.0017). A 1-way repeated-measure ANOVA was conducted to measure the effect of SVFL on OKN gain. There was a significant effect of SVFL F_(3.85,57.76)_ = 56.82, *P* < 0.0001 (nb fractional degrees of freedom result from Greenhouse-Geisser correction). The change in the mean OKN gain values was captured with the Naka-Rushton fit parameters (*R*_max_ = 1.16, *L*_50_ = 0.12, n = 0.49, goodness of Naka-Rushton fit: *R*^2^ = 0.94). Although the rightmost large-gray circle - corresponding to the full-field condition appears to be above the Naka-Rushton fit, it is not an outlier (z = 0.93, n = 16, *P* = 0.3524). The deviation of the mean gain from the Naka-Rushton expected value for the full field condition was 0.61 × SD (Fig. 4).

### Experiment 2: Effect of Simulated Contrast Loss on OKN Gain Across Two Visual Field Extents

**Figure 5.**
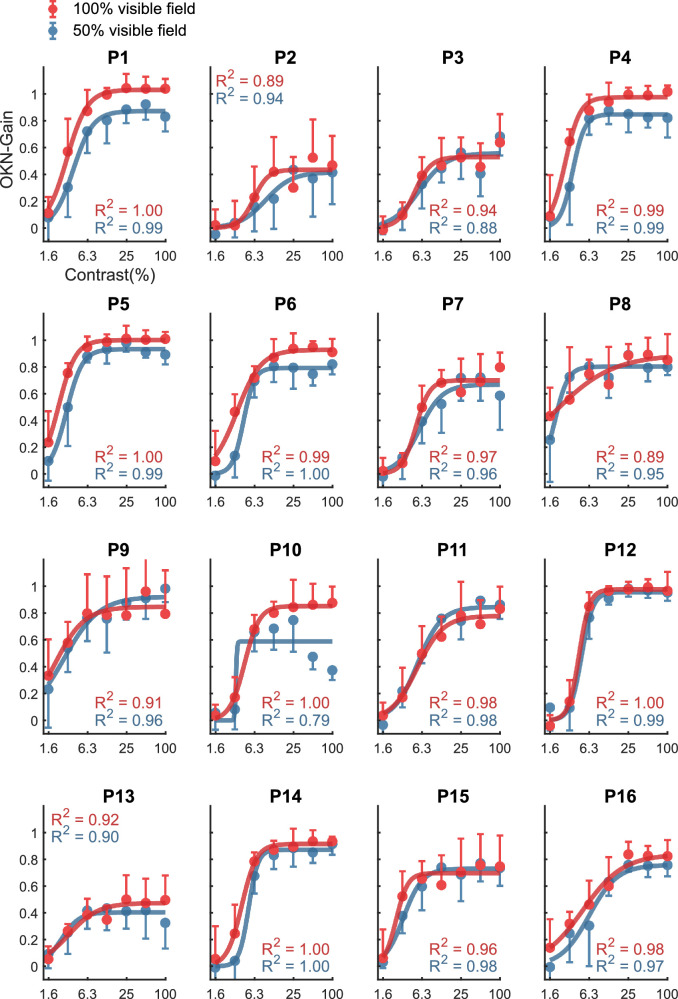
OKN gain plot against stimulus contrast for all sixteen participants. Red is full field and blue is 50% field. Data have been fitted with a Naka-Rushton function. Error bars span ±1 SD around the mean.

Figure 5 plots the OKN gain against % of stimulus contrast for our 16 participants. We note considerable variation both in the maximum OKN-response obtained and in the rate of increase of OKN, as a function of stimulus contrast. The Naka-Rushton fits on individual data revealed the *R*_max_ values of 0.75 ± 0.18 and 0.80 ± 0.19 (mean ± SD) and the C50 values of 4.4% ± 1.9% and 3.7% ± 1.3% for 50% and full field condition, respectively.

The OKN gain was correlated with log contrast levels of the stimulus ranging from 0.06 ± 0.09 at 1.6% contrast to 0.73 ± 0.21 at full contrast in the 50% field condition (*r* = 0.88, *P* = 0.010); and ranging from 0.10 ± 0.13 to 0.83 ± 0.17 for the full-field condition (*r* = 0.90, *P* = 0.006). Two-way repeated measure ANOVA was conducted to measure the effect of contrast across two VF extents on OKN gain. Although there were significant effects of contrast F_(2.14,32.07)_ = 120.50, *P* < 0.0001, and of visual field extent F_(1.00,15.00)_ = 14.59, *P* = 0.002, there was no interaction between contrast and VF extent F_(6,60)_ = 1.22, *P* = 0.30.

**Figure 6.**
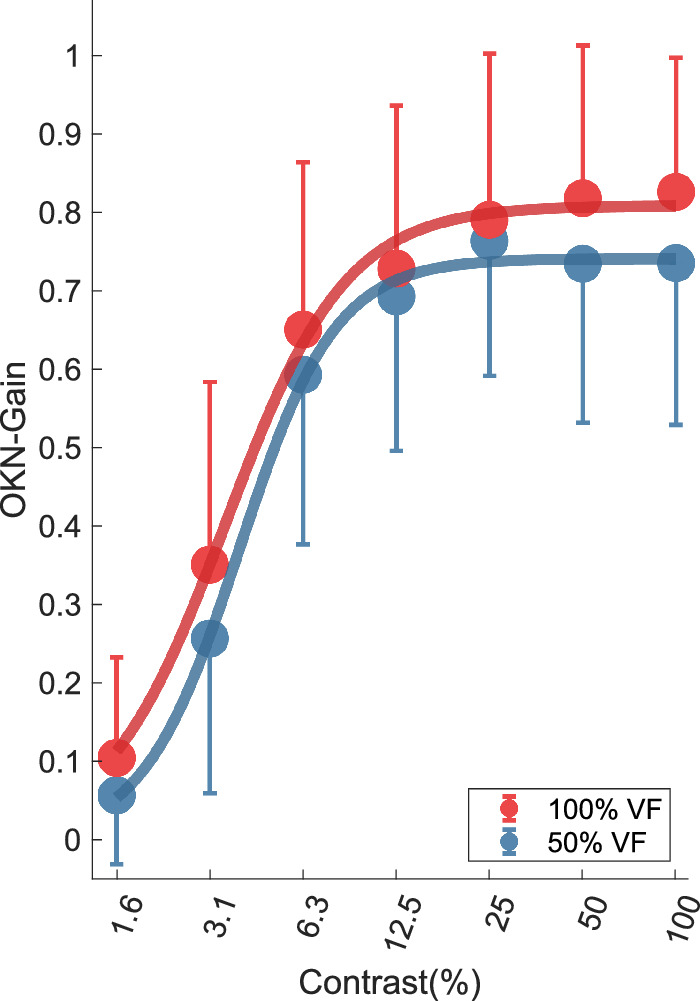
Results averaged across participants; the effect of contrast on OKN-gain for 2 visual field extents. OKN gain increases with contrast across 50% (*blue*) and full field (*red*) conditions. Error bars show 1 SD, but for clarity only the positive and the negative error bars are printed for the full field and 50% field conditions, respectively.

The *R_max_* parameter of the Naka-Rushton fit increased from 0.74 to 0.81 between the 50% and full-field conditions. In contrast, the *L*_50_ parameter remained constant at 3.89% and 3.52% for the 50% and 100% field conditions, respectively. *R*-squared values for the Naka-Rushton fits were *R^2^* = 0.997 and *R*^2^ = 0.995 for 50% field and full field conditions, respectively (Fig. 6).

**Figure 7.**
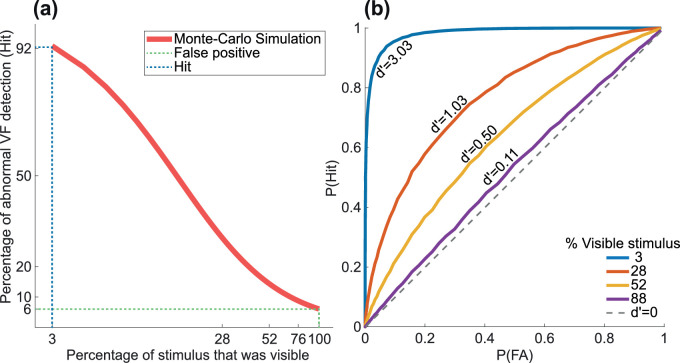
(a) Monte-Carlo simulation based on 100000 simulated subjects, showing percentage of successful detection of an abnormal visual field versus the extent of VF (on a log scale). (b) ROC curves and corresponding d’ values, here, calculated for 3, 28, 52 and 88% percent of stimulus that was visible.

The sensitivity and specificity of our OKN-gain based algorithm for signaling visual field loss was estimated using a Monte-Carlo simulation (100,000 repeats) with data from Experiment 1 (Fig. 7a). When the extent of VF was 100%, a cutoff criterion of mean OKN gain − 2 × SD yielded sensitivity of 92%, and when the VF extent was 3%, the specificity was 94%. The value of d′ was greater for higher degrees of VF loss (Fig. 7b).

## Discussion

Our results show that the gain of involuntary optokinetic nystagmus correlates with both simulated peripheral visual field loss (in Experiment 1), and with the contrast of the stimulus (in Experiment 2). The results of Experiment 1 show that OKN gain reduces as the extent of simulated VF decreases. Our results reveal a similar trend to earlier studies, which show that reducing the area of retinal stimulation using both open^28^ and closed-loop masks^36^ decreases the gain of OKN. In the open loop design, the OKN gain dropped from 0.86 for the full field condition to 0.70 for the 5-deg. width central stimulus.^28^ With the closed-loop design the OKN gain dropped from 0.9 for the full field condition to 0.77 for a 20-deg. square central stimulus.^36^ Although in our study the OKN gain for the full field condition is comparable to previous studies (0.92), the gain elicited by a 5-deg. diameter central stimulus is about the half of what expected. This lower gain could be due to our use of a smaller, circular area of visible stimulus, compared to the vertical band used in other studies.^28^ The results of Experiment 2 showed a significant reduction of the OKN gain as a result of lowering the contrast. We also observed a significant effect of simulated VF on reducing OKN-gain across all contrast levels tested: simulated VF loss exacerbates the effect of contrast loss.

The between-subject variation of OKN-gain that we observed is substantial but within the range reported by previous studies.^28^^,^^40^^,^^48^^,^^49^ The standard deviation (σ) of the mean for the full field condition was 0.15, which agrees with previous studies. For the 5-deg. diameter central stimulus in our study σ = 0.20, which is lower than σ = 0.33 reported by the only previous study using an open loop 5-deg. central stimulus (0.33 in Van Die and Collewijn's^28^ study). The lower σ in our study could be due to using masks with blurred edges, preventing them being used as a fixation target.^50^

Factors that might contribute to variation in individual OKN gains include participants’ age, residual myopic refractive error and their level of attention. Age is unlikely to be a significant factor since it does not impact maximum eye velocity during the slow phase of OKN for stimulus velocities lower than ∼50 deg/s.^51^ We also think it is unlikely that residual myopic refractive error would significantly influence our results. Our stimuli were high contrast (100%) low spatial frequency (1.3 c/deg) patterns. Consequently the maximum residual myopic defocus present in our participants (0.50 and 0.75D) would have a negligible effect on their visibility. The anticipated sensitivity loss from myopic defocus would be only 30% (with −0.50D) or 50% (with −0.75D) so that our 100% contrast stimuli would remain 70 or 50 times their detection threshold, respectively.^52^ Finally, considering attention, Magnusson, Pyykko et al (1985),^53^ report that an auditory or vibration cue presented during presentation of OKN stimuli (drifting at 90 deg/s) could significantly increase mean OKN gain. While instructing patients to follow features of the stimulus (look OKN) creates more cortical activation compared to stare OKN,^54^ at the drifting velocity used in our study (10 deg/s), there was no difference in OKN-gain depending on whether participants were instructed to “look” or to “stare”.^55^ Conversely, participants selectively attending to superimposed masks could actually decrease OKN gain,^50^ which could explain the substantial drop in OKN-gain observed with a superimposed mask, even when it barely occludes the drifting carrier. Differences in selective attention to this feature likely contribute to the variation of OKN gain between participants.

To minimize any impact on OKN gain from participants fixating areas of the mask, we used an open-loop masking paradigm and blurred the edges of the masks. Because high-contrast mask-edges can reduce OKN gain at velocities above 15 deg/s,^56^ we used a lower velocity of 10 deg/s. Even so, we observe a substantial difference in the OKN gain elicited by the 88%-field and full-field stimuli (Fig. 4). This discrepancy between the measured and expected OKN gain (based on our otherwise-excellent Naka-Rushton fits) in the full field condition is likely attributable to the presence of the mask, which is absent in the full field condition. The most likely explanation for this effect is the single video-frame delay (a maximum of 17 ms) that was present between the collection of the eye position sample, and the drawing of the next gaze contingent mask on the screen. In terms of our overall pattern of results then, we would expect this to have systematically reduced OKN gain in all SVFL conditions. We also note that any latency of the simulated scotoma would reduce OKN and therefore underestimate any predicted impact of glaucomatous field loss on OKN. In short, our findings likely represent a conservative estimate of the impact of real glaucomatous field loss on involuntary eye movements.

In terms of the potential of this approach for quantifying visual field loss, there are potentially several advantages to an OKN technique over SAP. First, it provides the clinician with a single measure of the functional visual field, which makes monitoring of the progression simpler than SAP. Second, it is an objective measure, and as such it is likely to be less affected by patient capability and willingness to do the test. Third, the OKN paradigm requires no response or judgment on patients’ part, which should make it more acceptable by them. Finally, administration of the OKN test requires less expertise, compared with SAP and electrophysiological methods. All of these factors could potentially contribute to a reduction of the variability of VF assessment using this method.

Given the limited impact of SVFL on OKN—recall a drop from 100% to 52% simulated visual field reduces gain by only 18%—is it likely that we will be able to base a test for quantifying glaucomatous vision loss on OKN? Loss of vision in the early stages of glaucoma does not only manifest as visual field loss. For example, patients that are pre-symptomatic (i.e. SAP indicates their visual fields are intact) may exhibit contrast sensitivity loss.^57^^–^^60^ Such a loss of vision in early stage glaucoma may result from ganglion cell dysfunction rather than drop-out which could potentially contribute to OKN reduction. Indeed a preliminary report has been made that OKN gain is lower in pre-symptomatic patients (i.e. who have normal fields according to SAP) than controls under some conditions.^61^ We have also recently measured OKN gain in 41 patients with asymmetric visual field loss (a minimum 10% difference in visual field index across the eyes) from primary open angle glaucoma. We report that differences between the OKN gain of the better eye and the worst eye are correlated with differences in visual field index (for a 12.5% contrast pattern, *r* = 0.61, *P* < 0.0001).^62^

However, a residual limitation on using the OKN technique is that it only gives an overall estimate of the functional visual field, with no topography. Spatial input to OKN is unlikely to be uniform across the peripheral field but localized binary m-sequence stimulation and spatial averaging (similar to multifocal electroretinography) could be used to obtain topographical maps.

In conclusion, OKN shows promise for the objective measurement of peripheral visual function. Further testing in patients with organic field loss is required to determine whether it will have utility in the clinic as an objective measure of field loss arising from conditions such as glaucoma.
